# Prognostic Impact of Type 2 Diabetes in Metastatic Colorectal Cancer

**DOI:** 10.7759/cureus.33916

**Published:** 2023-01-18

**Authors:** Mafalda Miranda Baleiras, Tiago Dias Domingues, Eduardo Severino, Carolina Vasques, Maria Teresa Neves, André Ferreira, Leonor Vasconcelos de Matos, Filipa Ferreira, Helena Miranda, Ana Martins

**Affiliations:** 1 Department of Medical Oncology, Centro Hospitalar de Lisboa Ocidental, Lisbon, PRT; 2 Centre of Statistics and its Applications (CEAUL), Faculdade de Ciências da Universidade de Lisboa, Lisbon, PRT; 3 Breast Unit, Centro Clínico Champalimaud, Lisboa, PRT

**Keywords:** metformin, hyperinsulinemia, insulin resistance, prognosis, metastatic colorectal cancer, diabetes mellitus

## Abstract

Background

Diabetes mellitus (DM) is a prognostic factor for some malignancies, but its clinical implications in metastatic colorectal cancer (mCRC) patients are less clear. Therefore, we conducted a retrospective study to evaluate the impact of pre-existing type 2 diabetes mellitus (T2DM) on the survival outcomes of patients with newly diagnosed mCRC.

Methodology

We retrospectively included patients with newly diagnosed mCRC between January 2017 and June 2021 and with pre-existing T2DM. Data on the characteristics of patients, clinicopathological features, and drug exposure were collected from the electronic medical records. The primary endpoint was overall survival (OS). Secondary endpoints were progression-free survival (PFS) and treatment-related adverse events (TRAEs).

Results

Among 187 mCRC patients, 54 (28.8%) had T2DM. The median follow-up was 25 months. We observed 150 OS events and 168 PFS events. Diabetes significantly and negatively impacted PFS and OS. The median for PFS (mPFS) was eight and 16 months for T2DM and no T2DM patients, respectively (p < 0.0001; log-rank test). The median overall survival (mOS) was 15 and 29 months for T2DM and no T2DM patients, respectively (p < 0.0001; log-rank test). Patients with diabetes were more often overweight or obese (59.3% vs. 24.8%; p < 0.01) and had a poorer performance status (53.7% vs. 21.1% with Eastern Cooperative Oncology Group Performance Status 1; p < 0.01). Additionally, T2DM patients had more high-risk pathological features, including G3 grading tumors (27.7% vs. 12.0%; p = 0.01), lymph node involvement (p < 0.01), *BRAF*-mutated (35.1% vs. 6.8%; p < 0.01), and right-sided CRC (63.0% vs. 30.1%; p < 0.01). We found no statistically significant differences in TRAEs. Nevertheless, a significantly higher rate of grade 2-4 peripheral neuropathy (22.2% vs. 5.3%; p < 0.01) was reported in T2DM patients.

Conclusions

T2DM is a negative prognostic factor for survival in mCRC. The paper provides empirical evidence in favor of the joint control of both pathologies. Further research is needed to establish the robustness of our results.

## Introduction

Diabetes mellitus (DM) is a growing worldwide concern, with a considerable impact on human life and healthcare costs. The global prevalence of diabetes in 2019 was estimated to be 9.3% (463 million people), rising to 10.2% (578 million) by 2030 and 10.9% (700 million) by 2045 [1]. Portugal has one of the highest prevalence rates (14.2% in 2020) in adults aged 20 to 79 years [1]. According to the Global Burden of Disease data, over one million deaths per year can be attributed to diabetes, making it the ninth leading cause of mortality [2].

Type 2 diabetes mellitus (T2DM) is the most common type of diabetes, representing 90% of all cases worldwide [1]. Several studies have described an association between T2DM and colorectal cancer (CRC) [3].

CRC is the third most common cancer worldwide (10.2%) and the second leading cause of cancer-related deaths (9%) [3]. Metastases are the main cause of CRC‐related mortality [4]. Approximately 15-30% of patients present with metastases at the time of initial diagnosis [5]. Metastatic colorectal cancer (mCRC) has poor outcomes, with a relative five‐year survival rate of 14% [4].

The association between T2DM and CRC has been partly attributed to common risk factors such as a sedentary lifestyle, a Western diet, obesity, and metabolic syndrome [6]. In addition, there are other potential pathophysiological and molecular mechanisms linking these two pathologies. Hyperinsulinemia, insulin-like growth factor (IGF) axis, hyperglycemia, chronic inflammation, and oxidative stress, which are biophysical characteristics of T2DM, play a key role in carcinogenesis. These mechanisms may explain not only the increased risk of CRC but also its worse prognosis in patients with a prior diagnosis of T2DM [3,7]. A recent analysis, including 80,193 patients with gastrointestinal cancer, showed that DM prevalence was the highest in patients with colon (15.5%) or rectal (15.3%) cancer [8]. Furthermore, hyperglycemia enhances resistance to 5-fluorouracil-induced apoptosis. Interestingly, various studies have shown a protective effect of metformin against cancer [9]. Metformin is an antidiabetic drug that decreases insulin resistance.

While there is more evidence of the association between diabetes and a higher risk of disease recurrence and death in localized CRC, the clinical implications of T2DM in mCRC are less clear [10]. Therefore, we conducted a retrospective study to evaluate the survival impact of pre-existing T2DM on the outcomes of patients with newly diagnosed mCRC.

## Materials and methods

Study design

We conducted a single institutional retrospective and observational study to evaluate the prognostic role of T2DM in mCRC. The study included all newly diagnosed mCRC patients between January 2017 and June 2021 at our Oncology Department in Hospital São Francisco Xavier, Lisbon, Portugal. This study was approved by the Institutional Ethics Committee in accordance with the Declaration of Helsinki.

Patient eligibility

We included patients with previously untreated mCRC confirmed by pathology and imaging according to the American Joint Committee on Cancer (AJCC) stage IV guidelines. Patients were aged 18 and older, with an Eastern Cooperative Oncology Group Performance Status (ECOG-PS) of 0-1. The hepatic, renal, and hematologic laboratory values were within the normal range. Patients with another primary neoplasia history, those with type 1 diabetes, and those under insulin administration were excluded. Patients with insufficient data about tumor pathological features or treatment received were also excluded.

Diabetes assessment

At the time of enrolment into the study, we documented physician-diagnosed history of T2DM from the medical records.

Study endpoints

The primary endpoint was overall survival (OS), which was assessed from the diagnosis of mCRC until death from any cause. Secondary endpoints were progression-free survival (PFS) and treatment-related adverse events (TRAEs). Patients without reported death were censored at their last known follow-up in our consultation. PFS was defined as the time from assignment until the first documented evidence of tumor, censor date, or death from any cause. Tumor progression was assessed by Response Evaluation Criteria in Solid Tumors (RECIST) 1.1. Adverse events were assessed by the treating physician or another doctor from our Department. TRAEs were graded according to the Common Terminology Criteria for Adverse Events (CTCAE) version 4.0. Peripheral neuropathy was included if grade ≥2 and all other adverse events if grade ≥3.

Covariates

Patients’ medical electronic records and pathology reports were reviewed. Information on clinical, pathological, and treatment data were gathered according to pre-determined categories comprising gender, age, body mass index, ECOG-PS, presence of T2DM, use of metformin, date of mCRC diagnosis, primary tumor location, metastatic involvement, RAS, *BRAF *and microsatellite (MS) status at initial diagnosis, type of first-line chemotherapy, and the number of chemotherapy lines.

Statistical analysis

For continuous variables, the median and interquartile range are presented. The underlying normality of data was assessed using the Kolmogorov-Smirnov test with Lilliefors correction. For categorical variables, results are presented as n (%). The Mann-Whitney test was used to compare continuous variables in the case of two independent samples. For categorical variables, the chi-square test or Fisher’s exact test was used when applicable. Regarding the estimation of OS and PFS, the non-parametric Kaplan-Meier estimator was used. Comparisons between survival times for independent groups were performed using the log-rank test. All results with a p-value smaller than 0.05 were considered statistically significant. Data analysis was performed using the R software version 4.2.2.

## Results

Clinical characteristics of the study population

Overall, 187 mCRC patients were included in this study, of whom 54 (28.9%) had T2DM. The median age was 69 years (61-76, minimum-maximum), and genders were quite equally distributed (50.8% male; 49.2% female). Diabetic patients were more often overweight or obese (59.3% vs. 24.8%; p < 0.01) and had a poorer ECOG-PS (53.7% vs. 21.1% with ECOG-PS 1; p < 0.01). Additionally, participants with T2DM were more likely to have G3-grade tumors (27.7% vs. 12.0%; p = 0.01) and right-sided CRC (63.0% vs. 30.1%; p < 0.01). There was a significant association between lymph node involvement and T2DM (p < 0.01). RAS and *BRAF *status assessed on resected tumors or biopsies from metastases were available in 183 cases. The frequency of *BRAF *mutation was significantly higher in diabetic patients (35.1% vs. 6.8%; p < 0.01), while the frequency of *KRAS *mutation was similar in both groups (33.3% vs. 36.1%, p = 0.69). Baseline demographic and clinicopathological features according to diabetes status are depicted in Table 1.

**Table 1:** Baseline demographic and clinicopathological features of the study population. Results are expressed as n (%) or median (interquartile range). *: statistically significant value. T2DM = type 2 diabetes mellitus; BMI = body mass index; ECOG-PS = Eastern Cooperative Oncology Group Performance Status; cT = clinical staging of primary tumor according to American Joint Committee on Cancer; cN = clinical lymph node staging according to American Joint Committee on Cancer; MS = microsatellite

Characteristics	Whole population (n = 187)	No T2DM (n = 133)	T2DM (n = 54)	P-value
Age (years)	69.0 (60.0–77.8)	69.0 (60.0–76.0)	70.0 (61.3–77.8)	0.52
Gender, n (%)				
Male	95 (50.8)	69 (51.9)	26 (48.1)	0.76
Female	92 (49.2)	64 (48.1)	28 (51.9)	
BMI (kg/m^2^), n (%)				
Normal (18.5–24.9)	122 (65.2)	100 (75.2)	22 (40.7)	<0.01*
Overweight/Obese (≥25)	65 (34.8)	33 (24.8)	32 (59.3)	
Pretreatment ECOG-PS, n (%)				
0	130 (69.5)	105 (78.9)	25 (46.3)	<0.01*
1	57 (30.5)	28 (21.1)	29 (53.7)	
Metformin use, n (%)	31 (16.6)	0 (0.0)	31 (57.4)	<0.01*
Differentiation grade, n (%)				
G1	43 (23.0)	36 (27.1)	7 (13.0)	0.01*
G2	113 (60.4)	81 (60.9)	32 (59.3)	
G3	31 (16.6)	16 (12.0)	15 (27.7)	
Side of primary tumor, n (%)				
Left	113 (60.4)	93 (69.9)	20 (37.0)	<0.01*
Right	74 (39.6)	40 (30.1)	34 (63.0)	
cT, n (%)				
T1	35 (18.7)	30 (22.6)	5 (9.3)	0.07
T2	40 (21.4)	31 (23.3)	9 (16.7)	
T3	64 (34.2)	40 (30.1)	24 (44.4)	
T4	48 (25.7)	32 (24.0)	16 (29.6)	
cN, n (%)				
N0	47 (25.1)	43 (32.3)	4 (7.4)	<0.01*
N1	84 (44.9)	67 (50.4)	17 (31.5)	
N2	56 (30.0)	23 (17.3)	33 (61.1)	
KRAS status, n (%)				
Mutated	66 (35.3)	48 (36.1)	18 (33.3)	0.69
Wildtype	119 (63.6)	84 (63.2)	35 (64.8)	
Unknown	2 (1.1)	1 (0.7)	1 (1.9)	
BRAF status, n (%)				
Mutated	28 (15.0)	9 (6.8)	19 (35.1)	<0.01*
Wildtype	157 (83.9)	123 (92.5)	34 (63.0)	
Unknown	2 (1.1)	1 (0.7)	1 (1.9)	
MS status, n (%)				
Stable	162 (86.6)	120 (90.2)	42 (77.8)	0.022*
Unstable	17 (9.1)	7 (5.3)	10 (18.5)	
Unknown	8 (4.3)	6 (4.5)	2 (3.7)	

Association between type 2 diabetes mellitus, tumor burden, and physician selection of chemotherapy regimen

Because the initial distant metastasis pattern is a relevant prognostic factor, its association with T2DM was explored. Interestingly, T2DM patients were more susceptible to developing high metastatic involvement (more than two sites, 20.4% vs. 7.5%; p < 0.01). Similarly, oligo-metastatic disease was more frequent among non-diabetic individuals (67.7% vs. 35.2%; p < 0.01). Although not significant, patients with T2DM were less likely to receive oxaliplatin-based chemotherapy. Diabetes was associated with a smaller cumulative number of therapy lines (p = 0.02) (Table 2).

**Table 2:** Data regarding the treatment and tumor burden of the study population. Results are expressed as n(%) or median (interquartile range). *: Statistically significant value. T2DM = type 2 diabetes mellitus; CT = chemotherapy

Characteristics	Whole population (n = 187)	No T2DM (n = 133)	T2DM (n = 54)	P-value
Metastatic involvement, n (%)				
One site	109 (58.3)	90 (67.7)	19 (35.2)	<0.01*
Two sites	57 (30.5)	33 (24.8)	24 (44.4)	
> two sites	21 (11.2)	10 (7.5)	11 (20.4)	
First-line backbone CT, n (%)				
FOLFIRI	77 (41.2)	53 (39.8)	24 (44.4)	0.68
FOLFOX	86 (46.0)	62 (46.6)	24 (44.4)	0.91
Capecitabine	19 (10.2)	13 (9.8)	6 (11.1)	0.99
FOLFOXIRI	5 (2.6)	5 (3.8)	0 (0.0)	0.32
Number of CT lines, n (%)				
One	58 (31.0)	36 (27.1)	22 (40.7)	0.02*
Two	76 (40.6)	51 (38.4)	25 (46.3)	
Three	36 (19.3)	31 (23.3)	5 (9.3)	
More than three lines	17 (9.1)	15 (11.3)	2 (3.7)	
No	119 (63.6)	84 (63.2)	35 (64.8)	

Association between type 2 diabetes mellitus and treatment-related adverse events

To explore the possible role of higher toxicity in the prognostic power of T2DM, we analyzed the TRAEs and treatment exposure in both groups (Table 3). Curiously, no statistically significant differences were found. Nevertheless, a significantly higher rate of grade 2-4 peripheral neuropathy (22.2% vs. 5.3%; p < 0.01) was reported in T2DM patients. Diarrhea, neutropenia, and asthenia were the most frequent grade 3-4 adverse events (24 in diabetic patients, 16 in non-diabetic patients). There were no treatment-related deaths. Although not significant, T2DM patients experienced a higher rate of dose reduction (57.4% vs. 41.3%; p = 0.07). Similarly, no differences were found in either treatment delay (T2DM: 48.1%, no T2DM: 42.9%; p = 0.62) or treatment suspension (T2DM: 5.6%, no T2DM: 4.5%; p = 0.72).

**Table 3:** Adverse events according to diabetes status. Results are expressed as n(%). *: Statistically significant value. ^b^: Adverse events were included if they were graded ≥3, judged as being possibly, probably, or definitely related to treatment. The only exception was for peripheral neuropathy which was considered if graded ≥2. T2DM = type 2 diabetes mellitus

Adverse event^b^	No T2DM (n = 133)	T2DM (n = 54)	P-value
Hematologic, n (%)			
Neutropenia	8 (6.0)	3 (5.6)	>0.1
Anemia	4 (3.0)	0 (0.0)	0.33
Gastrointestinal, n (%)			
Diarrhea	10 (7.5)	8 (14.8)	0.21
Vomiting	4 (3.0)	2 (3.7)	>0.1
Mucositis	2 (1.5)	0 (0.0)	>0.1
Anorexia	4 (3.0)	3 (5.6)	0.41
Neurologic, n (%)			
Peripheral neuropathy	7 (5.3)	12 (22.2)	<0.01*
Others, n (%)			
Hypertension	4 (3.0)	4 (7.4)	0.23
Pain	4 (3.0)	5 (9.3)	0.12
Asthenia	6 (4.5)	5 (9.3)	0.30

Prognostic impact of type 2 diabetes mellitus in metastatic colorectal cancer patients

**Figure 1:**
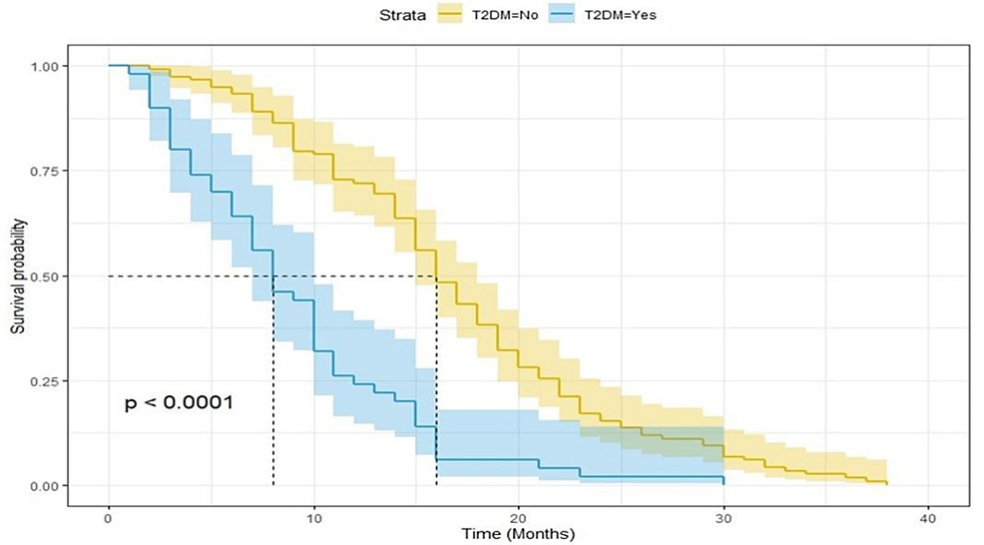
Kaplan-Meier curves for progression-free survival. T2DM = type 2 diabetes mellitus

**Figure 2:**
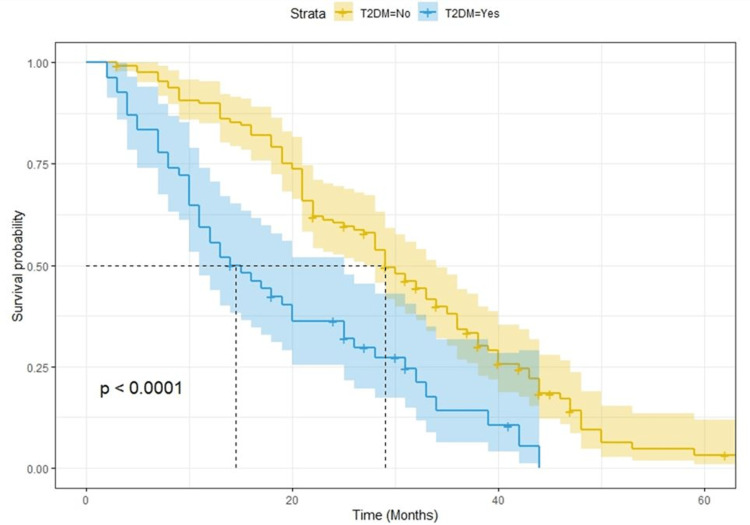
Kaplan-Meier curves for overall survival. T2DM = type 2 diabetes mellitus

**Figure 3:**
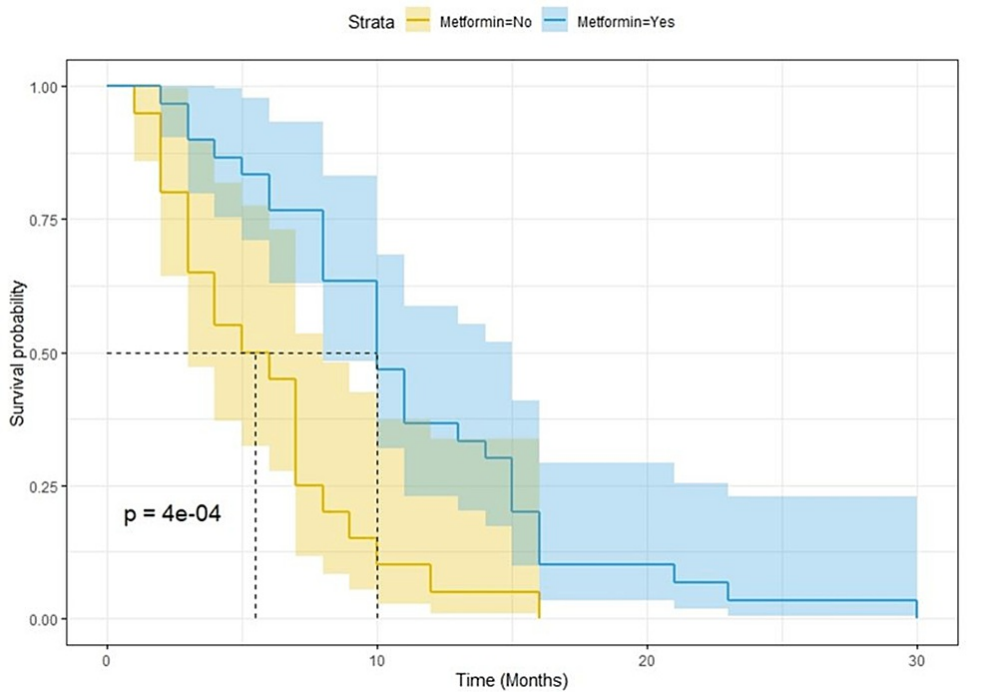
Kaplan-Meier curves for progression-free survival among T2DM patients according to metformin use. T2DM = type 2 diabetes mellitus

**Figure 4:**
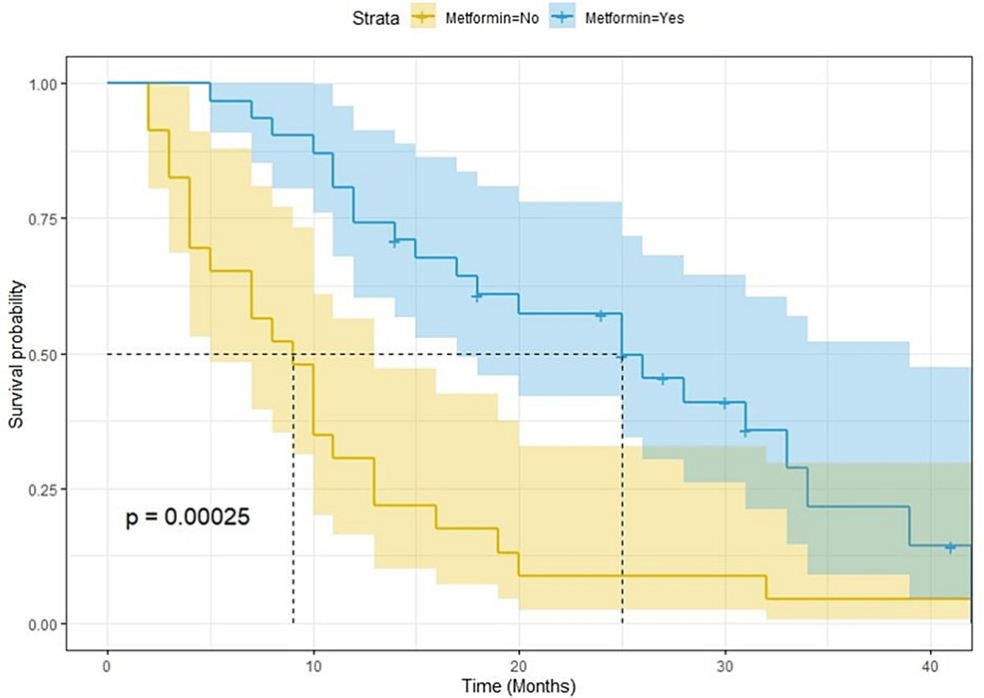
Kaplan-Meier curves for overall survival among T2DM patients according to metformin use. T2DM = type 2 diabetes mellitus

After a median follow-up of 25 months, there were 168 progression events and 150 deaths. Kaplan-Meier curves for PFS and OS showed a clear detrimental effect of T2DM (Figure 1 and Figure 2, respectively). The median survival for PFS was eight and 16 months for T2DM and no T2DM patients, respectively (p < 0.0001; log-rank test). The median overall survival was 15 and 29 months for T2DM and no T2DM patients, respectively (p < 0.0001; log-rank test). When analyzing the potential effect of metformin use among diabetic patients, we found out that PFS and OS were better for patients who were on metformin (Figure 3 and Figure 4, respectively). Median OS was 25 and nine months (p = 0.00025; log-rank test) and median PFS was 10 and five months for metformin and non-metformin users (p = 0.00004; log-rank test), respectively.

## Discussion

The main aim of this study was to examine the prognostic role of T2DM in mCRC patients. DM has been consistently reported to be an independent risk factor for CRC [11-13]. In a meta-analysis of 15 studies, Larsson et al. (2005) pointed out that individuals with diabetes had a relative risk for CRC of 1.30 (95% confidence interval (CI) = 1.20-1.40) compared with non-diabetic individuals [14]. However, most studies combine patients with metastatic and non-metastatic CRC and often do not distinguish between type 1 and type 2 diabetes.

Although various theories between CRC and T2DM have been proposed, the determinants of such linkages remain largely unknown [3,6,7]. Hyperinsulinemia secondary to insulin resistance, a central feature in diabetes, may play an important role in colorectal carcinogenesis. Insulin has been shown to increase the growth of colon epithelial and carcinoma cells in vitro [15]. In addition, elevated circulating levels of insulin increase bioactive IGF-I which, in turn, can induce cell proliferation and differentiation and inhibit apoptosis [16].

With an overall reported prevalence of 14.8% among all digestive tract cancer patients, diabetes is a public health issue [1,8]. Among 80,193 patients with gastrointestinal cancer, Roderburg et al. (2022) showed that DM prevalence was the highest in patients with colon (15.5%) or rectal (15.3%) cancer [8]. In our analysis, 54 patients (28.8%) had pre-existing T2DM. This difference might be explained by our smaller sample size, but also by the fact that Portugal has one of the highest prevalence rates of diabetes.

In this study, although not statistically significant, patients with T2DM were older. Consistent with our study, Chen et al. (2010) reported that diabetic patients were on average 5.3 years older compared with non-diabetic patients [17]. Furthermore, on comparing tumor locations, we found that proximal colon cancer (CC) predominated in the T2DM group. Xiao et al. (2022) conducted a systematic review and meta-analysis of 10 studies to examine the site-specific association between diabetes and the risk of CC. Among 17,624 patients, diabetes was associated with an increased risk of right-sided colon cancer (RSCC) compared with no diabetes (relative risk (RR) = 1.35; 95% CI = 1.24-1.47) [18]. RSCC is generally associated with poor prognosis and does not respond well to conventional chemotherapy [19]. Moreover, the diabetic patients in our study also presented with more poorly differentiated cancer and lymph node involvement. These patients can present with high-risk pathological features, including T3 and T4 tumors, which were also observed. A recent study also pointed out this association between T2DM and metastases to lymph nodes (p < 0.01) [20].

*BRAF *mutations, found in 8-12% of CRC cases, are usually associated with a poor prognosis. They tend to occur in proximal CC, in older patients, and often co-occur with high-level microsatellite instability [21]. Harlid et al. (2022), in a pooled analysis including 9,756 CRC patients, confirmed that diabetes particularly increases the risk of *BRAF*-mutated tumors [21].

CRC patients with diabetes are at a greater risk for all-cause and cancer-specific mortality and have worse disease-free survival compared with those without diabetes [22]. Subgroup analysis of two meta-analyses based on six studies revealed a 32% increase in all-cause mortality associated with diabetes in patients with CRC (95% CI = 1.24-1.41) [23]. In a later study published in 2013, a meta-analysis of 21 studies, pre-existing diabetes was associated with a 17% increased risk of all-cause mortality in CRC patients [22]. A possible explanation for this association has previously been attributed to the general effects of diabetes on mortality, including increased death from cardiovascular diseases. As such, in general, diabetes prevention is an important clinical recommendation to the population. Moreover, diabetic CRC patients are expected to benefit from both diseases if effective control of diabetes is achieved.

Focusing on cancer-specific mortality, data are inconclusive. While some studies found no significant correlation [23], others showed that CRC patients with diabetes had a 12% increased risk of cancer-specific mortality (RR = 1.12; 95% CI = 1.01-1.24) compared with those without diabetes. Although a similar trend was observed for cancer recurrence, this association did not reach statistical significance (RR = 1.24, 95% CI = 0.99-1.55) [22]. Several factors have been implicated in this association. First, some studies suggest that diabetic patients tend to receive less aggressive cancer treatment and experience a lower response rate to treatments compared with those without diabetes [22,24]. A possible explanation may be linked to underlying diabetes-related comorbidities that influence clinical decision-making, worse functional status (as seen in our sample), or higher treatment-related toxicities in diabetic patients.

A second possibility, as previously mentioned, is that increased levels of insulin or IGF may influence tumor aggressiveness. Various observations have shown that through those mechanisms tumor cell proliferation and angiogenesis can occur. Yet, the link between hyperinsulinemia and CRC mortality remains unclear.

Metformin, an oral biguanide agent, is known as the first-line antidiabetic agent for T2DM. It reduces insulin resistance and decreases blood glucose concentration by inhibiting gluconeogenesis and suppressing hepatic glucose production with improved peripheral tissue insulin sensitivity [25]. In addition to its antidiabetic effect, in vitro and in vivo reports have shown that metformin may have anticancer effects. They have shown that metformin inhibits cancer cell proliferation, metabolism, and angiogenesis by activating adenosine monophosphate-activated protein kinase and inhibiting the mammalian target of rapamycin signaling [25]. Moreover, while lowering systemic glucose levels and improving secondary hyperinsulinemia, metformin prevents the latter effects on tumor growth and progression. However, its antineoplastic effect in CRC is quite controversial [9].

A large Danish register-based study was conducted among diabetic CRC patients to examine the effect of metformin on CRC survival. Fransgaard et al. (2018) found no association between metformin use and recurrence-free or disease-free survival [26]. However, only patients treated medically for diabetes were included and classified as diabetic. On the contrary, Cheng et al. (2020) demonstrated better overall and cancer-specific survivals among diabetic metformin users with CRC [25]. Nevertheless, the authors acknowledged several limitations of their meta-analysis, including the heterogeneity between the studies, in terms of differences in CRC stage and length of follow-up as well as limited adjustment for confounding variables. Similarly, we identified 54 patients with pre-existing T2DM and stage IV CRC, of whom 57.4% (n = 31) were defined as metformin users. We found these patients had better survival than those not receiving metformin, which endorses the potential anticancer effects of metformin in CRC patients. Further studies to evaluate this link are warranted.

There are some limitations of this study. First, its single-institution retrospective nature is a limitation. The diagnosis of T2DM was based on a chart review, patients’ history, and medication use. Therefore, a proportion of patients may be undiagnosed with T2DM. Second, patients with diabetes often have additional comorbidities, such as cardiovascular and pulmonary diseases, which are independently prognostic of clinical outcomes. These additional health conditions were not assessed in our analysis. We did not state the cause of death, which would add important information to understanding the prognostic link between DM and CRC mortality. Causes of death were likely more related to cardiovascular disease than to metastasis of CRC. We lack this information. Lastly, we had a limited sample size, especially in T2DM patients (only 54 patients). More in-depth studies are needed to establish the robustness of our results.

## Conclusions

Our findings suggest that T2DM is not only a comorbidity but also a poor prognostic factor, increasing the risk of mortality and tumor progression among patients with mCRC. Moreover, diabetic patients with mCRC also have high-risk pathological features associated with a worse prognosis. Our results add evidence in favor of a combined therapy to control diabetes and CRC progression. This study underscores the usefulness of further research to understand the physiologic mechanisms underpinning the relationship between the two pathologies to improve outcomes in patients with T2DM and CRC.
